# Resuscitation and survival time in terminal cancer patients at the end of life: A retrospective observational study in a Chinese hospital

**DOI:** 10.1097/MD.0000000000046130

**Published:** 2025-11-21

**Authors:** Zhongping Yao, Hongxia Tan, Yuehe Tu, Shaowen Wang, Lixin Qu, Xiang Li

**Affiliations:** a Radiotherapy Department Ⅱ, Jinshazhou Hospital of Guangzhou University of Chinese Medicine, Guangzhou, Guangdong, China; b Center for Reproductive Medicine, Guangdong Second Provincial General Hospital, Guangzhou, Guangdong, China; c Fifth Department of Oncology, Jinshazhou Hospital of Guangzhou University of Chinese Medicine, Guangzhou, Guangdong, China; d Intervention Center, Jinshazhou Hospital of Guangzhou University of Chinese Medicine, Guangzhou, Guangdong, China.

**Keywords:** cardiopulmonary resuscitation (CPR), end-of-life care, intensive care unit (ICU), survival analysis, terminal cancer

## Abstract

End-of-life care decisions for terminal cancer patients remain a clinical and ethical challenge, particularly regarding the use of aggressive interventions. This retrospective observational study aimed to evaluate the impact of different treatment schemes on survival time in terminal cancer patients. A total of 1266 patients were categorized into 4 groups: Group A received mechanical assistance and other rescue measures in the intensive care unit (ICU); Group B received drug rescue with cardiopulmonary resuscitation in the general ward; Group C received drug rescue only; and Group D received no rescue treatment. Overall survival was estimated using Kaplan–Meier analysis, and between-group differences were assessed with stratified log-rank tests. The median survival times were: Group A: 138.0 hours (95% confidence interval [CI]: 109.1–166.8), Group B: 54.5 hours (95% CI: 42.8–66.3), Group C: 60.0 hours (95% CI: 51.7–68.3), and Group D: 60.4 hours (95% CI: 53.9–66.8). Group A showed significantly longer survival than Groups B, C, and D (*P* < .05), whereas no significant difference was observed among Groups B, C, and D (*P* > .05). ICU-based resuscitation may provide modest survival benefit for terminal cancer patients, whereas drug-based rescue and cardiopulmonary resuscitation outside the ICU do not appear to extend survival. These findings support individualized, evidence-based decision-making for end-of-life interventions.

## 1. Introduction

Cancer remains the 2nd leading cause of death globally, with a growing incidence and mortality rate. In 2018, cancer accounted for 9.55 million deaths worldwide, marking an increase of 720,000 from 2014 figures.^[1,2]^ While much oncological research has focused on prolonging life and improving long-term survival, significantly less attention has been paid to short-term survival and quality of death, particularly in the terminal phase of illness. Recent studies emphasize the importance of understanding patients’ preferences for end-of-life care and what constitutes a “good death” from their perspective. Although technological advances have improved cancer prognoses, critical gaps remain in aligning end-of-life care with patient values, especially regarding the intensity of treatments administered in the final days of life.^[3]^ Key elements such as decision-making, treatment preferences, and appropriate use of emergency interventions (e.g., ICU admission, cardiopulmonary resuscitation [CPR], and aggressive pharmacologic therapy) remain insufficiently addressed.

International palliative care models, particularly from Western countries, highlight the risks of overtreatment and advocate for patient-centered approaches that prioritize quality of life over life extension. Recent large-scale analyses continue to demonstrate that aggressive interventions at the end of life, such as ICU admissions, late chemotherapy, and emergency department visits, are frequently associated with reduced quality of death, greater emotional burden on families, and elevated healthcare costs. A 2024 global meta-analysis involving 129 studies found that approximately 14.4% of terminal cancer patients were admitted to the ICU within 30 days of death, 11.6% received chemotherapy in the final 14 days, and nearly 18% had multiple hospitalizations, indicating a continued reliance on aggressive care despite palliative alternatives.^[4]^ Similarly, a 2023 U.S. cohort study of over 146,000 older adults with metastatic cancer found that aggressive end-of-life care, including frequent hospitalizations and in-hospital death, remained common, particularly among nursing home residents.^[5]^ In contrast, studies consistently show that early initiation of palliative care (more than 3 months before death) is associated with fewer intensive interventions, better alignment with patient preferences, and improved end-of-life experiences.^[6]^

Despite this, there is a relative paucity of empirical research, especially in non-Western settings, examining how specific emergency treatments affect survival duration and end-of-life experiences in terminal cancer patients. One U.S. study involving 17,609 cancer patients found considerable variation in end-of-life care based on physicians’ preferences, often resulting in overtreatment.^[7]^ These findings underscore the need for a clearer understanding of how different treatment strategies impact dying trajectories and quality of death.

This study aims to retrospectively analyze patients with terminal malignancies to examine the relationship between various emergency clinical interventions (such as ICU admission, CPR, and drug use) and their outcomes in terms of survival time and characteristics of death. The goal is to identify patterns that can guide more appropriate, patient-centered care at the end of life.

## 2. Patients and methods

### 2.1. Patients

#### 2.1.1. Data source

This retrospective study analyzed inpatient medical records from Jinshazhou Hospital, Guangzhou University of Chinese Medicine, covering patients discharged between January 1, 2015, and December 31, 2022. Ethical approval was obtained from the Ethics Committee of Jinshazhou Hospital of Guangzhou University of Chinese Medicine (Guangzhou, China). All participants or their legal representatives provided written informed consent.

**Figure 1.**
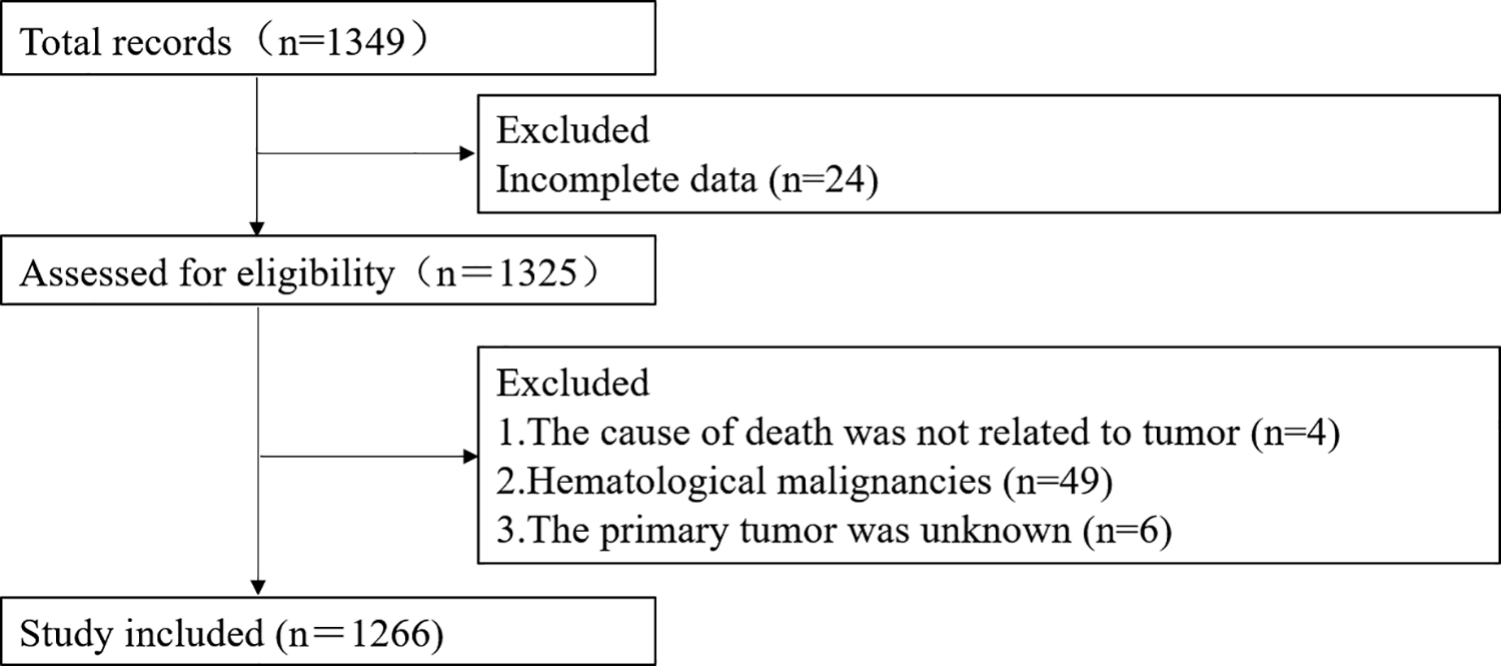
Patient enrollment screening process. Flowchart depicting the inclusion and exclusion of patients based on eligibility criteria. A total of 1266 patients were enrolled after applying exclusion criteria such as death outside the hospital, incomplete medical records, non-cancer-related causes of death, or doctor–patient disputes.

A total of 1266 patients were enrolled (790 males and 476 females) after applying the following exclusion criteria: death outside the hospital or abnormal death within the hospital; incomplete or missing medical records; deaths unrelated to cancer (i.e., direct or indirect non-cancer causes); and involvement in doctor–patient disputes. (see Fig. 1 for patient selection flowchart.)

### 2.2. Study design

This was a single-center, retrospective observational study, based on electronic medical records. The study period for each patient began with the physician’s notification of severe illness requiring rescue treatment and ended at the time of death. Criteria for “severe illness” included any of the following: respiratory failure requiring mechanical ventilation; neurological symptoms such as altered mental status, seizures, or coma; renal failure necessitating dialysis; digestive system emergencies requiring intensive care; severe infections such as sepsis or pneumonia; and critical trauma or poisoning (e.g., skull or vertebral fractures, drug or alcohol overdose).

Patients were classified into 4 groups based on the rescue strategies used: Group A: mechanical assistance (e.g., ventilators) and other intensive measures, including CPR, administered in the ICU; Group B: drug-based rescue (e.g., dopamine, epinephrine, lobeline) with CPR if needed, administered in a general ward; Group C: drug rescue only, without CPR; and Group D: no rescue treatment provided.

### 2.3. Endpoints and assessments

The primary endpoint was survival time from the notification of severe illness to death. Between-group comparisons were made to evaluate the impact of different treatment intensities. Subgroup analyses were also conducted to explore survival differences.

### 2.4. Statistical analysis

Survival analyses were performed using the Kaplan–Meier method, with differences assessed using stratified log-rank tests. Hazard ratios (HRs) and 95% confidence intervals (CIs) were calculated via a stratified Cox proportional hazards model. Proportional hazards assumptions were tested using Schoenfeld residuals. Multicollinearity among covariates was evaluated using variance inflation factor analysis. Censoring occurred at the time of discharge if the patient did not die in hospital, though such cases were rare due to inclusion criteria focusing on in-hospital deaths. (IBM SPSS version 25.0, Guangzhou, China) and (GraphPad Prism version 9.0, GraphPad Software, Guangzhou, China) were used for all statistical analyses. A 2-tailed *P*-value < .05 was considered statistically significant.

## 3. Results

A total of 1266 patients were included in the analysis, consisting of the following cancer types: gastrointestinal tumors (n = 321), hepatobiliary tumors (n = 217), lung cancers (n = 390), gynecological tumors (n = 119), head and neck tumors (n = 121), urinary system tumors (n = 60), and other tumors (n = 38) (Table 1). Patients were categorized into 4 treatment groups based on the level of rescue intervention received (Table 2): Group A: mechanical ventilation and intensive rescue (e.g., CPR in ICU); Group B: drug-based rescue with CPR in a general ward; Group C: drug-only rescue without CPR; and Group D: no rescue treatment.

**Table 1:** Tumor types and composition of enrolled patients.

	Tumor type	N	Total
Gastrointestinal neoplasms	Bowel cancer	162	321
	Gastric cancer	87	
	Esophageal carcinoma	32	
	Pancreatic cancer	40	
Hepatobiliary tumor	Liver cancer	191	217
	Gallbladder carcinoma	12	
	cholangiocarcinoma	14	
Lung cancer	Lung cancer	390	390
Gynecological tumor	Breast cancer	57	119
	Cervical cancer	30	
	Ovarian cancer	24	
	Endometrial carcinoma	6	
	Vulvar carcinoma	2	
Head and neck tumor	Nasopharyngeal carcinoma	42	121
	Laryngeal carcinoma	8	
	Thyroid carcinoma	3	
	Brain cancer	31	
	Mandibular carcinoma	2	
	Tonsil carcinoma	2	
	Buccal carcinoma	5	
	Carcinoma of the floor of the mouth	4	
	Parotid carcinoma	2	
	Tongue cancer	9	
	Oropharyngeal carcinoma	6	
	Gingival carcinoma	3	
	Ocular carcinoma	1	
	Hypopharyngeal carcinoma	3	
Tumor of the urinary system	Prostate cancer	31	60
	Renal carcinoma	18	
	Adrenal carcinoma	1	
	Bladder cancer	10	
Other types	Skin cancer	4	38
	Cardiac cancer	2	
	Peritoneal carcinoma	6	
	Thymic carcinoma	1	
	Pleural carcinoma	2	
	Bone cancer	6	
	Pelvic tumor	1	
	Sarcoma of chest	4	
	Mediastinal tumor	12	

Distribution of enrolled patients (n = 1266) according to primary cancer type, including gastrointestinal, hepatobiliary, lung, gynecological, head and neck, urinary system, and other malignancies.

**Table 2:** Number of patients enrolled and median age in each group.

Group	A	B	C	D
N	202 (16%)	236 (19%)	362 (28%)	466 (37%)
Age (yr)	65.0 ± 13.89	66.0 ± 16.17	68.0 ± 15.53	65.5 ± 14.76

Number of patients and corresponding median age in each of the 4 treatment groups: Group A: mechanical ventilation and intensive care rescue, Group B: pharmacologic rescue and CPR in general ward, Group C: pharmacologic rescue only, and Group D: no rescue treatment.

**Figure 2.**
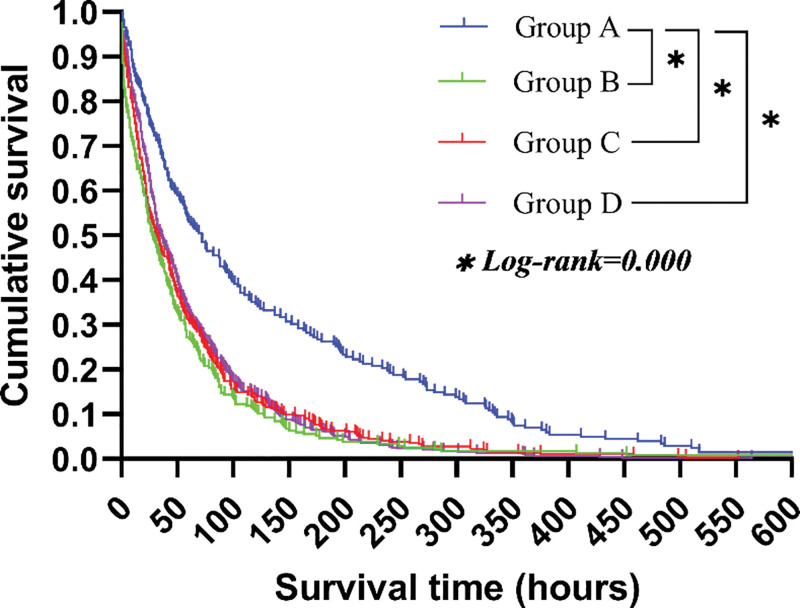
Kaplan–Meier survival curves comparing the 4 rescue treatment groups. Group A (mechanical ventilation and ICU-level care) demonstrated significantly longer survival time compared with Groups B, C, and D (log-rank *P* < .05). No significant differences were observed among Groups B, C, and D (*P* > .05). Risk tables could not be incorporated into the plots due to software limitations.

The median survival time from physician notification of critical illness to death was: Group A: 138.0 hours (95% CI: 109.1–166.8), Group B: 54.5 hours (95% CI: 42.8–66.3), Group C: 60.0 hours (95% CI: 51.7–68.3), and Group D: 60.4 hours (95% CI: 53.9–66.8). Kaplan–Meier analysis demonstrated that patients in Group A had significantly prolonged survival compared with Groups B, C, and D (log-rank *P* < .05). However, no significant differences were observed among Groups B, C, and D (*P* > .05) (Fig. 2).

**Figure 3.**
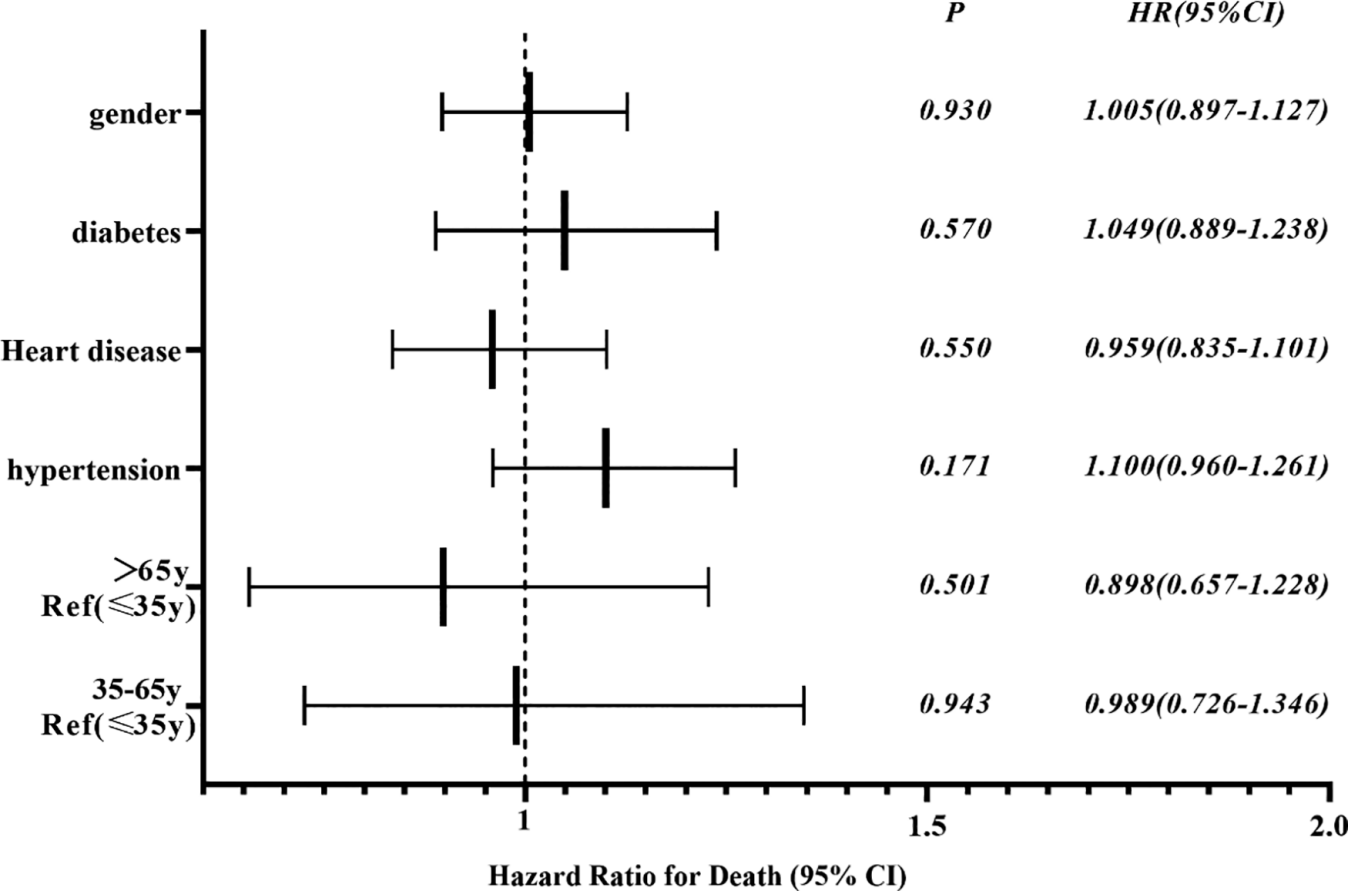
Forest plot of subgroup analysis for survival predictors. Subgroup analysis examining the effect of sex, age, and comorbidities (diabetes mellitus, hypertension, heart disease) on survival time from the onset of critical illness to death. No variable showed a statistically significant association with survival time (all *P* > .05).

Subgroup analyses were performed to evaluate whether age, sex, or comorbidities (e.g., diabetes mellitus, hypertension, heart disease) were associated with survival duration. None of these variables were found to be statistically significant predictors of survival time (all *P* > .05; Fig. 3).

## 4. Discussion

### 4.1. Definition of a good death

The concept of a “good death” is inherently multifaceted, shaped by personal values, cultural beliefs, and social context. For many terminally ill patients, maintaining autonomy and dignity, particularly through shared decision-making about the timing, setting, and mode of death, is central to achieving a death aligned with their values. Prior research has identified key components of a good death, including symptom control, comfort, emotional closure, respect for personal values, minimization of burden on family members, and effective communication with healthcare providers.^[8–10]^ In the Chinese cultural background, strong emphasis is placed on familial connection, dying at home, and maintaining dignity, often interpreted as avoiding visible suffering and invasive procedures.^[10,11]^ Accordingly, appropriate pain management and avoidance of unnecessary interventions are widely regarded as indicators of high-quality end-of-life care.^[12–14]^

Although studies from Western countries consistently show that most patients prefer to die at home prefer to die at home,^[15–18]^ systemic barriers, cultural expectations, and limited availability of hospice care in China often lead to prolonged hospital stays and aggressive treatments. Our findings echo this reality, highlighting that many Chinese cancer patients continue to receive intensive interventions even in the terminal phase of illness. Notably, patients often express a desire to remain communicative and spiritually whole until death, underscoring the need for humanistic, comfort-centered care over life-prolonging but burdensome treatments.

### 4.2. Treatment intention and the value of aggressive interventions

Despite global shifts toward palliative care models in oncology, aggressive end-of-life interventions, such as ICU admission, mechanical ventilation, and CPR, remain frequent, even in the absence of clear survival benefits.^[19–24]^ Our study found that ICU-based care (Group A) was associated with longer survival compared to other groups. However, as this was an observational study, the difference must be interpreted cautiously, and the associations observed should not be taken as evidence of causality, efficacy, or clinical benefit from ICU care. The survival benefit may reflect earlier or more aggressive supportive measures, yet our data do not account for baseline functional status, comorbidities, or severity of illness.^[25–27]^ Furthermore, patients who received CPR or drug-only interventions did not experience significant survival advantage compared to those who received no active resuscitative treatment, suggesting limited efficacy of such interventions in the final phase of illness.^[28–30]^

The importance of early palliative care integration is well established, with numerous studies demonstrating its role in improving symptom control, patient satisfaction, and even survival in some cases.^[31–35]^ For patients with advanced cancer and significant comorbidities, timely palliative care can reduce unnecessary hospitalizations and enhance quality of life.^[36]^

Interestingly, although most patients reportedly do not desire mechanical life support,^[12]^ ICU care is often initiated. A U.S.-based study similarly found minimal benefit from ICU interventions in terminal cancer patients,^[20]^ and the NCCN guidelines recommend minimizing CPR in patients with poor prognoses.^[37]^ However, in our cohort, 18.6% of patients elected for CPR, likely influenced by cultural attitudes toward hope and survival, as well as limited understanding of realistic outcomes. Previous studies have shown that when patients are presented with detailed prognostic data on CPR success, they are more likely to decline such interventions.^[38–40]^ The 2024 joint guidelines by the European Society of Intensive Care Medicine (ESICM) and the European Association for Palliative Care emphasize the importance of early goals-of-care discussions, culturally sensitive decision-making, and prioritization of comfort over life-prolonging measures when prognosis is poor.^[41]^ Similar to our results, another study demonstrated that aggressive interventions such as CPR and ICU admission were associated with reduced patient peacefulness, increased psychological distress for families, and a diminished sense of dignity in the final days of life.^[42]^ Moreover, systematic reviews highlighted the negative impact of inappropriate pharmacologic interventions, including excessive sedation, on both patients and caregivers, reinforcing the need to individualize end-of-life strategies and avoid reflexively escalated care.^[43,44]^

Nonetheless, discordance often exists between patient preferences and the decisions made by families or physicians who may be unaware of those preferences.^[45]^ A prior study also suggests that CPR does not meaningfully prolong life in terminally ill patients, but increases physical and emotional suffering.^[46]^ Consistent with previous reports, patients value comfort and communication ability in their final days,^[47]^ and many wish to delegate decision-making authority to a trusted family member or physician if they become incapacitated.^[48]^ These findings emphasize the importance of advance care planning and honest, compassionate conversations about prognosis and treatment goals.

### 4.3. The role of healthcare providers in end-of-life decisions

Physicians, particularly oncologists, often continue aggressive therapies such as chemotherapy and surgery late into the disease course, potentially influenced by a focus on disease-directed treatment and a reluctance to shift toward comfort care.^[20,49,50]^ Evidence indicates that treatment recommendations differ by specialty, with oncologists more likely to suggest discontinuation of treatment compared to surgeons.^[47]^ Moreover, prognostic accuracy is often poor,^[51]^ with many physicians overestimating survival in terminal patients,^[52–56]^ leading to inappropriate treatment plans and missed opportunities for hospice referrals.

Our findings align with the literature in suggesting that family members may request aggressive interventions due to limited understanding of prognosis, emotional distress, or a belief that continuing treatment equates to hope.^[57–59]^ This highlights the critical role of healthcare professionals in facilitating informed, values-based decisions. Effective communication about prognosis, patient preferences, and treatment burdens can help reduce unnecessary suffering and foster better outcomes.^[60]^

### 4.4. Evaluation of pharmacologic interventions at the end of life

Our data indicate that drug-based resuscitation or pharmacologic interventions did not significantly improve survival in terminally ill cancer patients. This aligns with international literature suggesting that, in end-stage illness, excessive medication use may not only lack efficacy but may also worsen the patient’s experience.^[61–65]^ Furthermore, non-pharmacologic aspects of care, such as psychological support, dignity preservation, and spiritual comfort, are often overlooked despite their recognized value to patients nearing the end of life.

### 4.5. Methodological considerations and limitations

This study has several important limitations. First, its retrospective and single-center design introduces inherent biases, including potential selection bias, as patient allocation to treatment groups was not randomized and may reflect institutional practices or clinician preferences. This limits the generalizability of our findings to broader patient populations or care settings. Second, the absence of randomization and multivariate adjustment limits our ability to draw causal inferences or account for key confounding variables such as baseline disease severity, age, comorbidities, and functional status. In addition, our dataset lacked complete information on cancer type-specific outcomes and patients’ do-not-resuscitate status, which may have influenced treatment decisions, survival outcomes, and the interpretation of subgroup analyses. These unmeasured factors may have influenced both treatment decisions and outcomes, potentially biasing our comparisons. Third, our dataset lacked comprehensive quality-of-life measures, patient-reported symptom assessments, and family-reported outcomes. These are essential to evaluating the full impact of end-of-life care, particularly in terms of comfort, dignity, and patient-centered values. Without these data, we were unable to assess the subjective experience of dying or the broader psychosocial consequences of aggressive interventions. Fourth, although survival time was used as the primary outcome, other important dimensions, such as symptom burden, psychological well-being, treatment-related distress, or quality of death, were not assessed, limiting our ability to evaluate the holistic impact of care. Finally, no formal power analysis was conducted. Although our sample size is comparable to other retrospective studies in this field, the study may have been underpowered to detect small but clinically meaningful differences between treatment groups.

## 5. Conclusion

This study found that among terminal cancer patients, only ICU-based medical rescue interventions were associated with a modest extension in survival time, while drug-based resuscitation and CPR provided no significant benefit. These findings highlight the need to critically evaluate the role of aggressive interventions in end-of-life care. Greater emphasis should be placed on aligning treatment decisions with patient preferences, improving communication with families, and integrating palliative care earlier in the disease trajectory. Reducing non-beneficial interventions can help minimize patient suffering and support a more dignified, value-consistent death. These insights have important implications for clinical practice guidelines and health policy aimed at optimizing end-of-life care.

## Author contributions

**Data curation:** Zhongping Yao, Shaowen Wang, Xiang Li.

**Formal analysis:** Hongxia Tan.

**Funding acquisition:** Shaowen Wang.

**Investigation:** Xiang Li.

**Resources:** Yuehe Tu.

**Software:** Hongxia Tan, Yuehe Tu.

**Supervision:** Lixin Qu.

**Writing – review & editing:** Lixin Qu, Xiang Li.

## References

[1] World Health Organization. Global Health Estimates (GHE). Available from: https://www.who.int/data/gho/data/themes/mortality-and-global-health-estimates. Accessed November 5, 2025.

[2] International Agency for Research on Cancer. World Cancer Report 2014. Lyon, France: IARC; 2014. Available from: https://publications.iarc.fr/Non-Series-Publications/World-Cancer-Reports/World-Cancer-Report-2014. Accessed November 5, 2025.

[3] Hughes T Schumacher M Jacobs-Lawson JM Arnold S. Confronting death: perceptions of a good death in adults with lung cancer. Am J Hosp Palliat Care. 2008;25:39–44. 18160544 10.1177/1049909107307377

[4] Ma Z Li H Zhang Y. Prevalence of aggressive care among patients with cancer near the end of life: a systematic review and meta-analysis. EClinicalMedicine. 2024;71:102561. 38549585 10.1016/j.eclinm.2024.102561 PMC10972834

[5] Koroukian SM Douglas SL Vu L. Incidence of aggressive end-of-life care among older adults with metastatic cancer living in nursing homes and community settings. JAMA Netw Open. 2023;6:e230394. 36811860 10.1001/jamanetworkopen.2023.0394 PMC9947721

[6] Heung Y Zhukovsky D Hui D Lu Z Andersen C Bruera E. Quality of end-of-life care during the COVID-19 pandemic at a comprehensive cancer center. Cancers. 2201;15:2201. 10.3390/cancers15082201 PMC10136926 37190130

[7] George LS Duberstein PR Keating NL. Estimating oncologist variability in prescribing systemic cancer therapies to patients in the last 30 days of life. Cancer (0008543X) 2024 Nov 1;130:3757–67 10.1002/cncr.35488 39077884

[8] Kehl KA. Moving toward peace: an analysis of the concept of a good death. Am J Hosp Palliat Care. 2006;23:277–86. 17060291 10.1177/1049909106290380

[9] Di Mola G Crisci MT. Attitudes towards death and dying in a representative sample of the Italian population. Palliat Med. 2001;15:372–8. 11591088 10.1191/026921601680419410

[10] Fairrow AM McCallum TJ Messinger-Rapport BJ. Preferences of older African-Americans for long-term tube feeding at the end of life. Aging Ment Health. 2004;8:530–4. 15724835 10.1080/13607860412331303829

[11] Li T Pei X Chen X Zhang S. Identifying end-of-life preferences among Chinese patients with cancer using the heart to heart card game. Am J Hosp Palliat Care. 2021;38:62–7. 32270684 10.1177/1049909120917361

[12] Steinhauser KE Christakis NA Clipp EC McNeilly M McIntyre L Tulsky JA. Factors considered important at the end of life by patients, family, physicians, and other care providers. JAMA. 2000;284:2476–82. 11074777 10.1001/jama.284.19.2476

[13] Mak JM Clinton M. Promoting a good death: an agenda for outcomes research – a review of the literature. Nurs Ethics. 1999;6:97–106. 10358525 10.1177/096973309900600202

[14] Proulx K Jacelon C. Dying with dignity: the good patient versus the good death. Am J Hosp Palliat Care. 2004;21:116–20. 15055511 10.1177/104990910402100209

[15] Townsend J Frank AO Fermont D. Terminal cancer care and patients’ preference for place of death: a prospective study. BMJ. 1990;301:415–7. 1967134 10.1136/bmj.301.6749.415 PMC1663663

[16] Gomes B Higginson IJ Calanzani N. Preferences for place of death if faced with advanced cancer: a population survey in England, Flanders, Germany, Italy, the Netherlands, Portugal and Spain. Ann Oncol. 2012;23:2006–15. 22345118 10.1093/annonc/mdr602

[17] Boyce-Fappiano D Liao K Miller C Peterson SK Elting LS Guadagnolo BA. Greater preferences for death in hospital and mechanical ventilation at the end of life among non-whites recently diagnosed with cancer. Support Care Cancer. 2021;29:6555–64. 33913005 10.1007/s00520-021-06226-5 PMC8081562

[18] Mystakidou K Parpa E Tsilika E. Where do cancer patients die in Greece? A population-based study on the place of death in 1993 and 2003. J Pain Symptom Manage. 2009;38:309–14. 19329275 10.1016/j.jpainsymman.2008.09.007

[19] Earle CC Neville BA Landrum MB Ayanian JZ Block SD Weeks JC. Trends in the aggressiveness of cancer care near the end of life. J Clin Oncol. 2004;22:315–21. 14722041 10.1200/JCO.2004.08.136

[20] Earle CC Landrum MB Souza JM Neville BA Weeks JC Ayanian JZ. Aggressiveness of cancer care near the end of life: is it a quality-of-care issue? J Clin Oncol. 2008;26:3860–6. 18688053 10.1200/JCO.2007.15.8253 PMC2654813

[21] Wilkerson DH Santos JL Tan X Gomez TH. Too much too late? chemotherapy administration at the end of life: a retrospective observational study. Am J Hosp Palliat Care. 2021;38:1182–8. 33111535 10.1177/1049909120966619

[22] Zdenkowski N Cavenagh J Ku YC Bisquera A Bonaventura A. Administration of chemotherapy with palliative intent in the last 30 days of life: the balance between palliation and chemotherapy. Intern Med J. 2013;43:1191–8. 23870085 10.1111/imj.12245

[23] Cheung MC Earle CC Rangrej J. Impact of aggressive management and palliative care on cancer costs in the final month of life. Cancer. 2015;121:3307–15. 26031241 10.1002/cncr.29485 PMC4560956

[24] Wright AA Zhang B Keating NL Weeks JC Prigerson HG. Associations between palliative chemotherapy and adult cancer patients’ end of life care and place of death: prospective cohort study. BMJ. 2014;348:g1219. 24594868 10.1136/bmj.g1219 PMC3942564

[25] Emanuel EJ Young-Xu Y Levinsky NG Gazelle G Saynina O Ash AS. Chemotherapy use among medicare beneficiaries at the end of life. Ann Intern Med. 2003;138:639–43. 12693886 10.7326/0003-4819-138-8-200304150-00011

[26] Matsuyama R Reddy S Smith TJ. Why do patients choose chemotherapy near the end of life? A review of the perspective of those facing death from cancer. J Clin Oncol. 2006;24:3490–6. 16849766 10.1200/JCO.2005.03.6236

[27] Seow H Barbera L Sutradhar R. Trajectory of performance status and symptom scores for patients with cancer during the last six months of life. J Clin Oncol. 2011;29:1151–8. 21300920 10.1200/JCO.2010.30.7173

[28] Peppercorn JM Smith TJ Helft PR. American society of clinical oncology statement: toward individualized care for patients with advanced cancer. J Clin Oncol. 2011;29:755–60. 21263086 10.1200/JCO.2010.33.1744

[29] Smith TJ Schnipper LJ. The American society of clinical oncology program to improve end-of-life care. J Palliat Med. 1998;1:221–30. 15859832 10.1089/jpm.1998.1.221

[30] Hui D Kim YJ Park JC. Integration of oncology and palliative care: a systematic review. Oncologist. 2015;20:77–83. 25480826 10.1634/theoncologist.2014-0312 PMC4294615

[31] Van Lancker A Velghe A Van Hecke A. Prevalence of symptoms in older cancer patients receiving palliative care: a systematic review and meta-analysis. J Pain Symptom Manage. 2014;47:90–104. 23764109 10.1016/j.jpainsymman.2013.02.016

[32] Trajkovic-Vidakovic M de Graeff A Voest EE Teunissen SCCM. Symptoms tell it all: a systematic review of the value of symptom assessment to predict survival in advanced cancer patients. Crit Rev Oncol Hematol. 2012;84:130–48. 22465016 10.1016/j.critrevonc.2012.02.011

[33] Romano AM Gade KE Nielsen G. Early palliative care reduces end-of-life Intensive Care Unit (ICU) Use but Not icu course in patients with advanced cancer. Oncologist. 2017;22:318–23. 28220023 10.1634/theoncologist.2016-0227 PMC5344633

[34] May P Garrido MM Cassel JB. Prospective cohort study of hospital palliative care teams for inpatients with advanced cancer: earlier consultation is associated with larger cost-saving effect. J Clin Oncol. 2015;33:2745–52. 26056178 10.1200/JCO.2014.60.2334 PMC4550689

[35] May P Normand C Cassel JB. Economics of palliative care for hospitalized adults with serious illness: a meta-analysis. JAMA Intern Med. 2018;178:820–9. 29710177 10.1001/jamainternmed.2018.0750 PMC6145747

[36] May P Garrido MM Cassel JB. Palliative care teams’ cost-saving effect is larger for cancer patients with higher numbers of comorbidities. Health Aff (Millwood). 2016;35:44–53. 26733700 10.1377/hlthaff.2015.0752 PMC4849270

[37] National comprehensive cancer network: NCCN clinical practice guidelines in supportive care: palliative care (version 1.2023). https://www.nccn.org/professionals/physician_gls/pdf/palliative.pdf Accessed August 7, 2025

[38] O’Brien LA Grisso JA Maislin G. Nursing home residents’ preferences for life-sustaining treatments. JAMA. 1995;274:1775–9. 7500508

[39] Teno J Lynn J Connors AF Jr.. The illusion of end-of-life resource savings with advance directives. SUPPORT Investigators. Study to understand prognoses and preferences for outcomes and risks of treatment. J Am Geriatr Soc. 1997;45:513–8. 9100723 10.1111/j.1532-5415.1997.tb05180.x

[40] Frankl D Oye RK Bellamy PE. Attitudes of hospitalized patients toward life support: a survey of 200 medical inpatients. Am J Med. 1989;86:645–8. 2729313

[41] Kesecioglu J Rusinova K Alampi D. European Society of Intensive Care Medicine guidelines on end of life and palliative care in the intensive care unit. Intensive Care Med. 2024;50:1740–66. 39361081 10.1007/s00134-024-07579-1 PMC11541285

[42] Zhang B Nilsson ME Prigerson HG. Factors important to patients’ quality of life at the end of life. Arch Intern Med. 2012;172:1133–42. 22777380 10.1001/archinternmed.2012.2364 PMC3806298

[43] Lo JJ Graves N Chee JH Hildon ZJ. A systematic review defining non-beneficial and inappropriate end-of-life treatment in patients with non-cancer diagnoses: theoretical development for multi-stakeholder intervention design in acute care settings. BMC Palliative Care. 2022;21:195. 36352403 10.1186/s12904-022-01071-7 PMC9644578

[44] Cardona-Morrell M Kim JC Turner RM Anstey M Mitchell IA Hillman K. Non-beneficial treatments in hospital at the end of life: a systematic review on extent of the problem. Int J Qual Health Care. 2016;28:456–69. 27353273 10.1093/intqhc/mzw060

[45] Covinsky KE Fuller JD Yaffe K. Communication and decision-making in seriously ill patients: findings of the SUPPORT project. the study to understand prognoses and preferences for outcomes and risks of treatments. J Am Geriatr Soc. 2000;48:S187–93. 10809474 10.1111/j.1532-5415.2000.tb03131.x

[46] Phillips RS Wenger NS Teno J. Choices of seriously ill patients about cardiopulmonary resuscitation: correlates and outcomes. SUPPORT Investigators. study to understand prognoses and preferences for outcomes and risks of treatments. Am J Med. 1996;100:128–37. 8629646 10.1016/s0002-9343(97)89450-8

[47] Curtis JR Cook DJ Sinuff T. Noninvasive positive pressure ventilation in critical and palliative care settings: understanding the goals of therapy. Crit Care Med. 2007;35:932–9. 17255876 10.1097/01.CCM.0000256725.73993.74

[48] Puchalski CM Zhong Z Jacobs MM. Patients who want their family and physician to make resuscitation decisions for them: observations from SUPPORT and HELP. Study to understand prognoses and preferences for outcomes and risks of treatment. hospitalized elderly longitudinal project. J Am Geriatr Soc. 2000;48:S84–90. 10809461 10.1111/j.1532-5415.2000.tb03146.x

[49] Barnato AE Herndon MB Anthony DL. Are regional variations in end-of-life care intensity explained by patient preferences?: a study of the US medicare population. Med Care. 2007;45:386–93. 17446824 10.1097/01.mlr.0000255248.79308.41 PMC2147061

[50] von Gunten CF. Discussing hospice care. J Clin Oncol. 2003;21:31s–6s. 12743186 10.1200/JCO.2003.01.163

[51] Hui D Con A Christie G Hawley PH. Goals of care and end-of-life decision making for hospitalized patients at a canadian tertiary care cancer center. J Pain Symptom Manage. 2009;38:871–81. 19811887 10.1016/j.jpainsymman.2009.05.017

[52] Glare PA Eychmueller S McMahon P. Diagnostic accuracy of the palliative prognostic score in hospitalized patients with advanced cancer. J Clin Oncol. 2004;22:4823–8. 15570085 10.1200/JCO.2004.12.056

[53] Loprinzi CL Johnson ME Steer G. Doc, how much time do I have. J Clin Oncol. 2003;21:5s–7s. 12743178 10.1200/JCO.2003.01.155

[54] Lamont EB Christakis NA. Prognostic disclosure to patients with cancer near the end of life. Ann Intern Med. 2001;134:1096–105. 11412049 10.7326/0003-4819-134-12-200106190-00009

[55] Hui D Kilgore K Nguyen L. The accuracy of probabilistic versus temporal clinician prediction of survival for patients with advanced cancer: a preliminary report. Oncologist. 2011;16:1642–8. 21976316 10.1634/theoncologist.2011-0173 PMC3233300

[56] Glare P Virik K Jones M. A systematic review of physicians’ survival predictions in terminally ill cancer patients. BMJ. 2003;327:195–8. 12881260 10.1136/bmj.327.7408.195 PMC166124

[57] Mack JW Cook EF Wolfe J Grier HE Cleary PD Weeks JC. Understanding of prognosis among parents of children with cancer: parental optimism and the parent-physician interaction. J Clin Oncol. 2007;25:1357–62. 17416854 10.1200/JCO.2006.08.3170

[58] Lee SJ Fairclough D Antin JH Weeks JC. Discrepancies between patient and physician estimates for the success of stem cell transplantation. JAMA. 2001;285:1034–8. 11209174 10.1001/jama.285.8.1034

[59] Laryionava K Hauke D Heußner P Hiddemann W Winkler EC. “Often relatives are the key […]” – Family involvement in treatment decision making in patients with advanced cancer near the end of life. Oncologist. 2021;26:e831–7. 33037846 10.1002/onco.13557 PMC8100569

[60] Mack JW Wolfe J Cook EF Grier HE Cleary PD Weeks JC. Hope and prognostic disclosure. J Clin Oncol. 2007;25:5636–42. 18065734 10.1200/JCO.2007.12.6110

[61] Ferrell B Virani R Grant M Juarez G. Analysis of palliative care content in nursing textbooks. J Palliat Care. 2000;16:39–47. 10802963

[62] Ferrell BR Virani R Grant M. Analysis of symptom assessment and management content in nursing textbooks. J Palliat Med. 1999;2:161–72. 15859813 10.1089/jpm.1999.2.161

[63] Rabow MW Hardie GE Fair JM McPhee SJ. End-of-life care content in 50 textbooks from multiple specialties. JAMA. 2000;283:771–8. 10683056 10.1001/jama.283.6.771

[64] Maddison AR Fisher J Johnston G. Preventive medication use among persons with limited life expectancy. Prog Palliat Care. 2011;19:15–21. 21731193 10.1179/174329111X576698 PMC3118532

[65] LeBlanc TW McNeil MJ Kamal AH Currow DC Abernethy AP. Polypharmacy in patients with advanced cancer and the role of medication discontinuation. Lancet Oncol. 2015;16:e333–41. 26149885 10.1016/S1470-2045(15)00080-7

